# Mechanosensitive protein polycystin-1 promotes periosteal stem/progenitor cells osteochondral differentiation in fracture healing

**DOI:** 10.7150/thno.93269

**Published:** 2024-04-08

**Authors:** Ran Liu, Yu-Rui Jiao, Mei Huang, Nan-Yu Zou, Chen He, Min Huang, Kai-Xuan Chen, Wen-Zhen He, Ling Liu, Yu-Chen Sun, Zhu-Ying Xia, L. Darryl Quarles, Hai-Lin Yang, Wei-Shan Wang, Zhou-Sheng Xiao, Xiang-Hang Luo, Chang-Jun Li

**Affiliations:** 1 Department of Endocrinology, Endocrinology Research Center, Xiangya Hospital of Central South University, Changsha, Hunan, 410008, China.; 2 Department of Medicine, University of Tennessee Health Science Center, Memphis, TN, 38163, USA.; 3 Department of Orthopaedics, The Second Affiliated Hospital of Fuyang Normal University, Fuyang, Anhui, 236000, China.; 4 Department of Orthopaedics, The First Affiliated Hospital of Shihezi University, Shihezi 832061, China.; 5 Key Laboratory of Aging-related Bone and Joint Diseases Prevention and Treatment, Ministry of Education, Xiangya Hospital, Central South University, Changsha, Hunan, 410008, China.; 6 National Clinical Research Center for Geriatric Disorders, Xiangya Hospital, Central South University, Changsha, Hunan, 410008, China.; 7 Laboratory Animal Center, Xiangya Hospital, Central South University, Changsha, Hunan, 410008, China.

**Keywords:** Polycystin-1, Periosteal Stem/Progenitor Cells, Fracture healing, Mechanical stress

## Abstract

**Background:** Mechanical forces are indispensable for bone healing, disruption of which is recognized as a contributing cause to nonunion or delayed union. However, the underlying mechanism of mechanical regulation of fracture healing is elusive.

**Methods:** We used the lineage-tracing mouse model, conditional knockout depletion mouse model, hindlimb unloading model and single-cell RNA sequencing to analyze the crucial roles of mechanosensitive protein polycystin-1 (PC1, *Pkd1*) promotes periosteal stem/progenitor cells (PSPCs) osteochondral differentiation in fracture healing.

**Results:** Our results showed that cathepsin (*Ctsk*)-positive PSPCs are fracture-responsive and mechanosensitive and can differentiate into osteoblasts and chondrocytes during fracture repair. We found that polycystin-1 declines markedly in PSPCs with mechanical unloading while increasing in response to mechanical stimulus. Mice with conditional depletion of *Pkd1* in *Ctsk^+^* PSPCs show impaired osteochondrogenesis, reduced cortical bone formation, delayed fracture healing, and diminished responsiveness to mechanical unloading. Mechanistically, PC1 facilitates nuclear translocation of transcriptional coactivator TAZ via PC1 C-terminal tail cleavage, enhancing osteochondral differentiation potential of PSPCs. Pharmacological intervention of the PC1-TAZ axis and promotion of TAZ nuclear translocation using Zinc01442821 enhances fracture healing and alleviates delayed union or nonunion induced by mechanical unloading.

**Conclusion:** Our study reveals that *Ctsk^+^* PSPCs within the callus can sense mechanical forces through the PC1-TAZ axis, targeting which represents great therapeutic potential for delayed fracture union or nonunion.

## Introduction

Bone fractures are a global public health issue. Approximately 160-190 million new bone fractures occur each year, and despite advances in surgical treatments, 5-10% of patients suffer from impaired healing 1, 2. Decreased loading stimuli, e.g., lack of mechanical loading in the setting of prolonged bed rest and paralysis, result in reduced bone healing capacity, leading to delayed fusion or nonunion 3-5. However, the underlying mechanism of mechanical stimuli regulating the fracture healing remains incompletely understood. Delineating how mechanical force regulates fracture healing is essential for developing new strategies for treating delayed fusion or nonunion.

Periosteal Stem/Progenitor cells (PSPCs), with multidirectional differentiation potential and self-renewal ability, play critical roles in fracture healing 6-11. Notably, an appropriate biomechanical environment is conducive to PSPC function and fracture healing 12. A moderate mechanical force promotes PSPC-mediated callus formation, whereas mechanical unloading leads to dysfunction of PSPCs, abnormal callus formation, and ultimately delayed union or nonunion 13-16. Genetic mouse models and lineage tracing techniques have identified several markers that label the PSPCs during bone fracture healing, including cathepsin K (*Ctsk*), *PDGFRα*, and *Prrx1* 8-10, 17-21. Recently, *Ctsk*-positive cells have been reported to be a primary source of PSPCs for cortical bone homeostasis and regeneration with the intramembranous and endochondral bone formation ability 10, 22. However, crucial questions remain about which type of PSPCs sense mechanical signals in the fracture callus microenvironment and how they convert mechanical signals into biological signals.

Advances in mechano-transduction have provided new insights into understanding how cells respond to the mechanical properties of the bone microenvironment 5, 23-26. Polycystin-1 (PC1), a large transmembrane protein encoded by the *Pkd1* gene, exhibits unique functions due to its extracellular domain that acts as a sensor to mechanical stimuli and an intracellular domain that forms a complex with PC2 and TAZ to transmit signals to the cell 27-30. Our previous studies have shown that PC1 is a key determinant of trabecular bone homeostasis 31-35. For example, PC1 plays an important role in osteogenesis by regulating the bone master transcription factor Runx2. Selective inactivation of *Pkd1* in the early stages of osteoblast lineage cells demonstrated impaired osteogenesis and a severe osteoporotic phenotype 36. Nevertheless, the role of PC1 in cortical bone homeostasis or bone regeneration is yet to be determined.

In this study, we provided evidence that *Ctsk^+^* PSPCs can sense mechanical stress and regulate osteochondrogenesis and bone repair through PC1. The presence of *Pkd1* in *Ctsk^+^* PSPCs is essential for maintaining cortical bone homeostasis and is indispensable for fracture healing. Conditional depletion of *Pkd1* in *Ctsk^+^* PSPCs showed decreased cortical thickness, impaired fracture healing, and diminished responsiveness to mechanical unloading. Mechanistic studies revealed that PC1 facilitates nuclear translocation of the transcriptional coactivator TAZ via PC1-CTT cleavage and plays a significant role in regulating the osteochondral differentiation potential of PSPCs. Applying a small molecule Zinc01442821, which specifically targets PC1-TAZ axis, improved the delayed union caused by mechanical unloading. This finding suggests that targeting this axis could be a promising therapeutic strategy for enhancing fracture healing in mechanical unloading.

## Results

### 1. *Ctsk^+^* cells are mechanosensitive PSPCs for fracture healing

We explored the mechanical regulation of bone fracture healing by analyzing the healing process in a fractured mouse model with hindlimb unloading (HU) at different time points post-fracture (Figure S1A). The mice of the HU group showed hardly discernible callus at 7- and 10-days post-fracture (dpf) and exhibited fracture nonunion at 20 dpf (Figure S1A). Moreover, compared to the ground group, the callus index (CI) was also decreased in the HU group (Figure S1B). Safranin O/Fast green staining indicated that cartilage formation was significantly impaired in HU mice, as evidenced by smaller islands of cartilage and a decreased woven bone area in the fracture site (Figure S1C-G). Although the bone area of HU group was comparable with that of the ground group, the HU group exhibited disordered bony callus on day 20 following the fracture, without evidence of successive bone callus formation (Figure S1C&D), suggesting that mechanical unloading induces delayed fracture repair.

**Figure 1:**
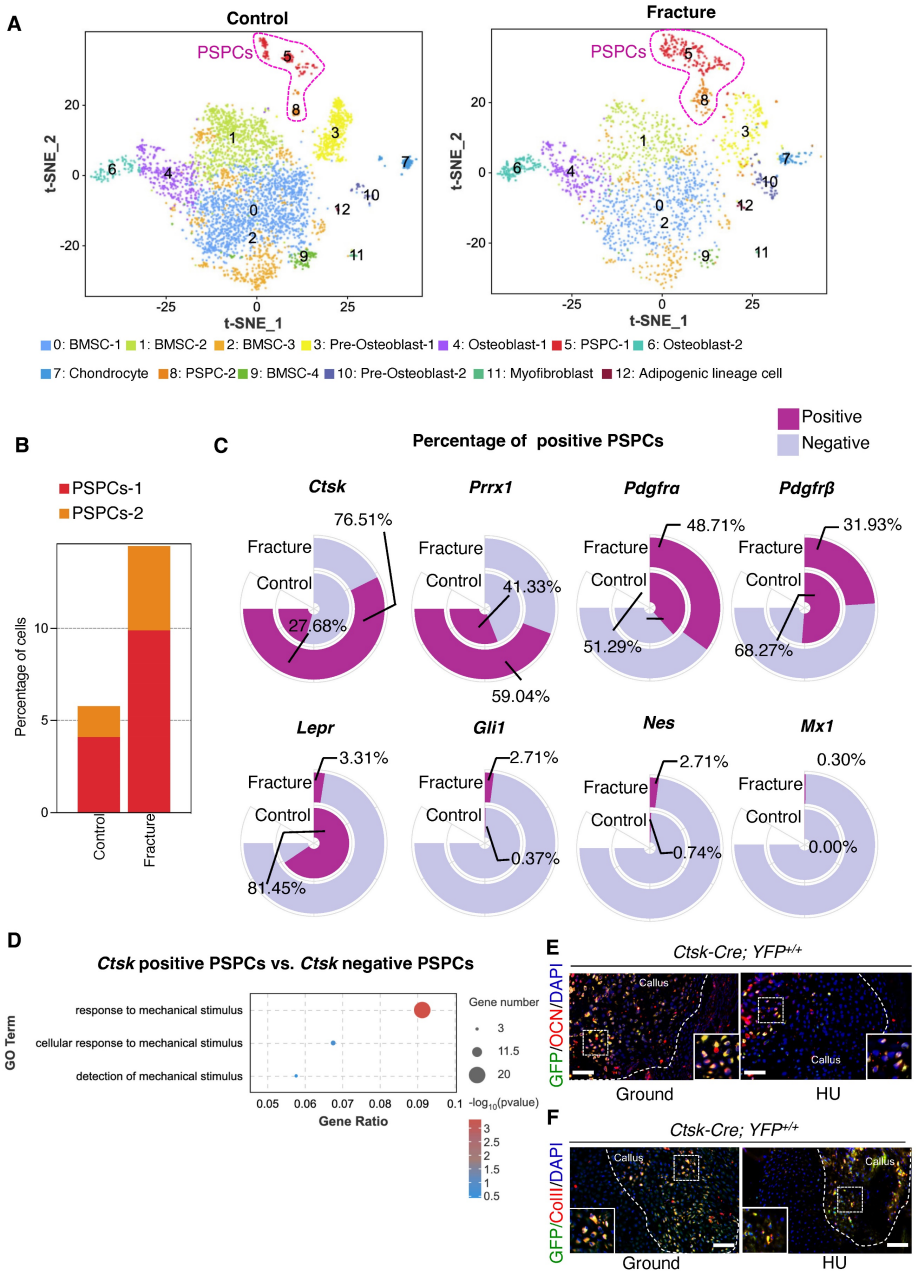
***Ctsk^+^* PSPCs are mechanosensitive with osteochondral differentiation potential and contribute to fracture healing. A** t-SNE plots showing 13 distinct clusters of cells identified and color-coded from mice fracture models (control group and fracture group). **B** Stacked bar chart showing the percentages of PSPCs within callus tissue quantified at 7 days post-fracture.** C** Circular stacked bar plot showing the proportion of positive or negative cells expressing markers gene of PSPCs in control group and fracture group. **D** GO analysis of differentially expressed genes in *Ctsk* positive PSPCs or *Ctsk* negative PSPCs related to mechanical stimuli. **E, F** Representative IF images of fracture callus at 14 days post-fracture in *Ctsk-Cre; YFP^+/+^* mice, immunostained with OCN (red) or COLLII (red) and GFP (green) antibodies and counterstained with DAPI (blue). White squares indicate magnified areas in callus. Scale bars = 100 μm. Data are presented as means ± SD. Unpaired t test.

PSPCs are identified as potential contributors for the skeletal regeneration process. We performed single-cell RNA sequencing (scRNA-seq) analysis on callus tissues in the fracture site 37 to determine the population and function of PSPCs in callus. We first characterized the changes in cell clusters from bone callus during fracture healing 7, 8, 10, 38, 39. A total of 13 cell clusters were recognized in the database (Figure 1A and Figure S2A). The cell types were annotated according to the expression of marker genes (Figure S2A-C). The PSPC population was more abundant in bone callus at 7 days post fracture compared with the control group (Figure 1B). This observation revealed that PSPCs in callus, responding early and quickly, were the primary source involved in forming the cartilaginous callus during fracture healing.

Subsequently, we compared the markers previously identified in labeling PSPCs, including *Ctsk*, *Prrx1, Pdgfrα*, *Pdgfrβ*, *LepR*,* Mx1, Nes,* and* Gli1* 6, 9, 10, 19, 40, 41. We found that the proportion of *Ctsk*-positive PSPCs was highest (76.51%) and increased dramatically in response to fracture (Figure 1C), implying that *Ctsk^+^* PSPCs may be the major source involved in fracture healing. Additionally, the results indicated that *Ctsk* expression is relatively high in PSPC-1 and PSPC-2, particularly in PSPC-1 (cluster 5) (Figure S2A, C). We performed Gene Ontology (GO) analysis and stemness, cell cycle and differentiation scores for PSPC-1&2, and the results showed that PSPC1 and 2 were involved in the similar biological functions following the fracture (Table S1 and Figure S3A-F). *Ctsk* was also expressed in pre-osteoblasts (cluster 3), osteoblasts (cluster 4 and cluster 6), and chondrocytes (cluster 7), which are all potentially derived from periosteal stem/progenitor cells (Figure S2A, C). Consistent with this observation, previous studies reported that *Ctsk-*labeled PSPCs were primarily involved in chondrogenesis and osteogenesis during fracture healing 10. To better comprehend the role of *Ctsk^+^* PSPCs in fracture healing, we compared differentially expressed genes (DEGs) in *Ctsk-*positive or *Ctsk-*negative PSPCs. GO analysis revealed that these DEGs were relevant in mechanical stimuli response, indicating that *Ctsk-*positive PSPCs may represent the predominant cell subset responding to mechanical stimulation following fracture (Figure 1D). Thus, we opted to utilize *Ctsk^+^* cells to investigate the effects of mechanical stimulus on PSPC function during fracture repair.

We established a *Ctsk-YFP* fluorescent tracing mouse (*Ctsk-Cre; YFP^+/+^*) to label and trace *Ctsk^+^* PSPC subpopulations in callus to examine further their functional changes in mechanical unloading during *in vivo* fracture healing. *In situ* fluorescence staining using antibodies against osteocalcin (OCN) revealed that OCN-positive GFP^+^ PSPCs were abundant in the callus of mice in the ground group but decreased significantly in the callus of mice with HU (Figure 1E, Figure S1H). Similarly, compared with the ground group, the HU group showed decreased type II collagen (ColII) fluorescence intensity in the *Ctsk*^+^ PSPCs (Figure 1F, Figure S1I). These data suggested that *Ctsk*^+^ PSPCs are mechanosensitive, and their osteogenic and chondrogenic ability is impaired by mechanical unloading during fracture healing.

### 2. Mechanical stimulus affects polycystin-1 level* in Ctsk^+^* PSPCs

**Figure 2:**
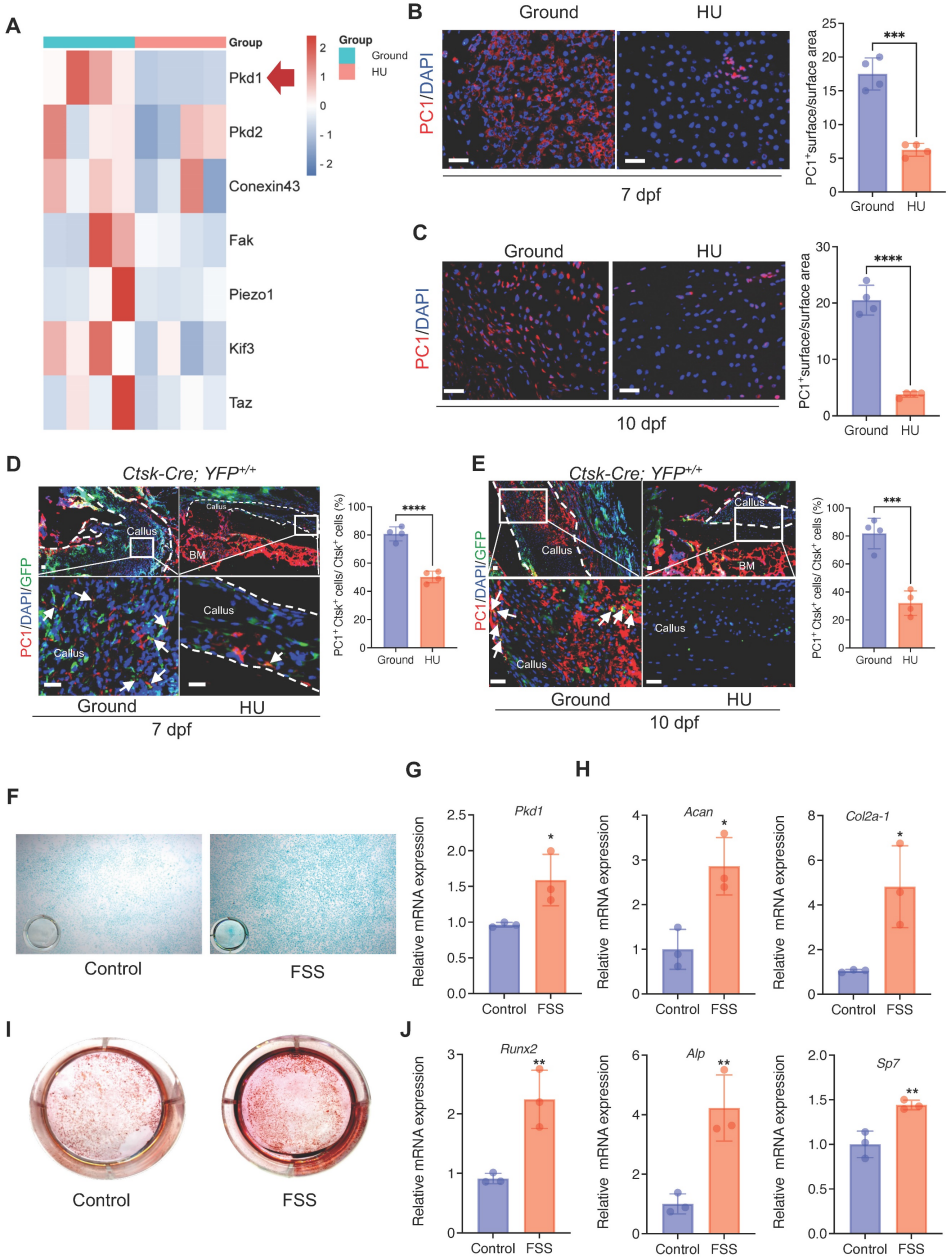
** Mechanical stimulus affects polycystin-1 level* in Ctsk^+^* PSPCs. A** Mechanosensitive gene expression levels in callus from fracture mice at 10 days post-fracture (n = 4). **B, C** Representative IF images of fracture callus at 7 days and 10 days post-fracture, immunostained with Ctsk (green) and PC1 (red) antibodies and counterstained with DAPI (blue). Dotted squares indicate areas magnified in Periosteum. Scale bars = 100 μm. Immunofluorescence intensity was quantified using ImageJ software (n = 4). **D, E** Representative IF images of fracture callus at 7- and 10-days post-fracture in *Ctsk-Cre; YFP^+/+^* mice, immunostained with Ctsk (green) and PC1 (red) antibodies and counterstained with DAPI (blue). White squares indicate magnified areas in callus. White arrows indicate co-localization of PC1 level in *Ctsk* positive cells. Immunofluorescence intensity was quantified using ImageJ software (n = 4). **F, I** Representative images of Alcian blue staining (F)and Alizarin Red staining (I) of PSPCs with fluid shear stress (FSS). **G, H, J** qPCR analysis of the efficiency of *Pkd1* gene knockout (G), chondrogenic-related genes expression (H) and osteogenic-related genes expression (J) in PSPCs with control and FSS treatment (n = 3). Scale bars = 100 μm. Data are shown as mean ± SD. *p < 0.05, **p < 0.01, ***p < 0.001 and **** p < 0.0001. ns, no significance.

The above data prompted us to test how *Ctsk^+^* PSPCs sense mechanical stimuli. We analyzed the mechanical sensing-related genes in bone callus tissue from the HU and control groups. We found that, besides *Pkd2*,* Fak*, and *Conexin43, Pkd1* (coding gene for polycystin-1, PC1) expression was substantially downregulated with mechanical unloading (Figure 2A). Meanwhile, immunofluorescence staining showed that HU induced a notable reduction in PC1 level in the bone callus on 7 and 10 dpf (Figure 2B&C). To determine the PC1 level further in *Ctsk* lineage cells under mechanical unloading, we performed PC1 immunofluorescence staining on the fractured callus of *Ctsk-Cre; YFP^+/+^* mice. We observed a significantly reduced PC1 level in *Ctsk*-positive cells in the callus areas in the HU-treated group compared to the ground group (Figure 2D, E).

To validate the direct influence of mechanical stress on the PSPC functions and PC1 level *in vitro*, we isolated primary PSPCs and subjected the cells to fluid shear stress (FSS). We found that the *Pkd1* level increased in the FSS-treated PSPCs (Figure 2G). The osteochondrogenic capacity of PSPCs was also enhanced along with increased expression levels of *Cola2-1*,* Acan* (chondrogenesis markers) (Figure 2F, H), and *Runx2, ALP,* and *Sp7* (osteogenesis markers) (Figure 2 I, J). These data showed PC1 level changes in PSPCs in response to different mechanical stimuli.

### 3. *Pkd1* deletion in *Ctsk^+^* PSPCs impairs cortical bone formation

**Figure 3:**
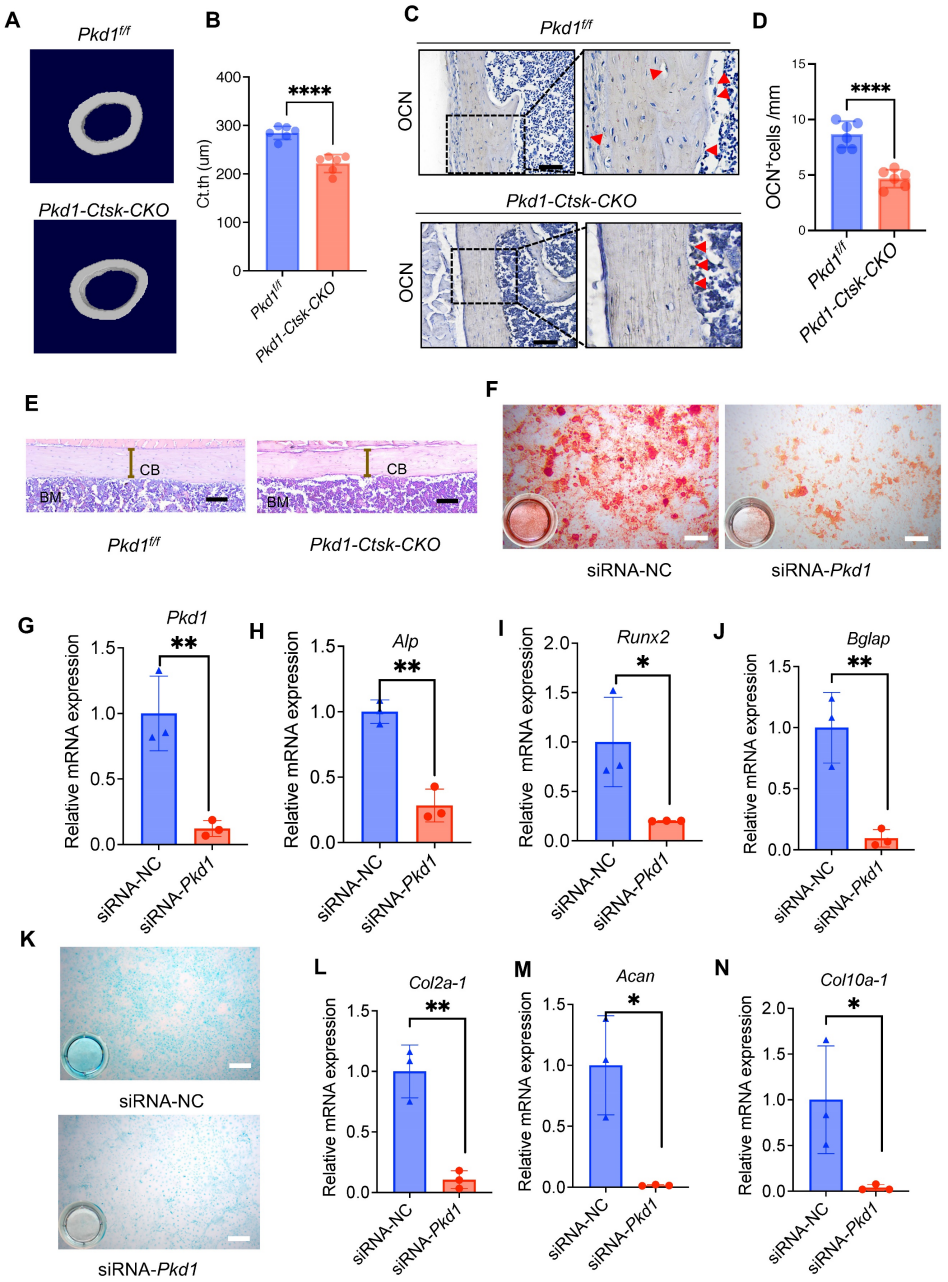
***Pkd1* deletion in *Ctsk^+^* PSPCs impairs bone formation. A, B** μCT images in femurs from 8-week-old male *Pkd1-Ctsk-CKO* mice (A) and quantitative analysis of the indicated parameters in *Pkd1-Ctsk-CKO* mice (B), respectively (n = 6). **C, D** Representative images of OCN immunohistochemical staining with analysis of number of osteogenic potentials of PSPCs (scalebar = 100 μm; n = 6). **E** Representative images of H&E staining with analysis of cortical thickness. Scale bars indicates 100 μm. **F** Representative images of Alizarin Red staining of PSPCs with transfection of siRNA-NC or siRNA-*Pkd1*. **G** qPCR analysis of *Pkd1* gene expression levels in PSPCs transfected with siRNA-NC or siRNA-*Pkd1* (n = 3). Scale bar indicates 100 μm. **H-J** qPCR analysis of osteogenic-related genes expression in PSPCs transfected with siRNA-NC or siRNA-*Pkd1* (n = 3). **K** Representative images of Alcian blue staining of PSPCs with transfection of siRNA-NC or siRNA-*Pkd1*. Scale bar indicates 100 μm. **L-N** qPCR analysis of chondrogenic-related genes expression in PSPCs transfected with siRNA-NC or siRNA-*Pkd1* (n = 3). Data are shown as mean ± SD. *p < 0.05, **p < 0.01 and **** p < 0.0001. ns, no significance.

We investigated the role of *Pkd1* in* Ctsk^+^* PSPCs function and cortical bone formation. For this, *Pkd1* gene was specifically ablated in *Ctsk^+^* PSPCs by crossing *Pkd1^flox/flox^* mice with *Ctsk-Cre* to generate *Pkd1-Ctsk-CKO* mice; *Pkd1^flox/flox^* mice were used as controls. Depletion of *Pkd1* in* Ctsk^+^* PSPCs resulted in a markedly thinner femoral cortex (Figure 3A, B). Furthermore, the depletion of *Pkd1* in* Ctsk^+^* PSPCs resulted in a reduced number of OCN-positive osteoblasts on the cortical bone surface (Figure 3C, D). H&E staining also confirmed that *Pkd1-Ctsk-CKO* mice had a lower cortical bone volume than the control group (Figure 3E). Since the *Ctsk* gene expression was previously reported in mature osteoclasts, we generated specific deletion of *Pkd1* gene in osteoclasts by crossing *Pkd1^flox/flox^* mice with *Trap-Cre.* The strategy excluded the potential contribution of PC1 depletion-mediated osteoclast functional changes in the bone phenotype of *Pkd1-Ctsk-CKO* mice. Contrary to the phenotype of reduced cortical bone thickness in *Pkd1-Ctsk-CKO* mice, *Pkd1-Trap-CKO* mice displayed significantly increased cortical bone thickness (Figure S4A, B). These results indicated that *Pkd1* in *Ctsk^+^* progenitor stem cells were indispensable for cortical bone formation and homeostasis, and specific depletion of *Pkd1* in* Ctsk^+^* PSPCs resulted in cortical thickness reduction.

We further validated the direct effect of *Pkd1* on PSPCs by transfecting *Pkd1* siRNA into PSPCs (Figure 3F), followed by the assessment of their osteochondrogenic characteristics. PSPCs transfected with *Pkd1* siRNA showed decreased expression of osteogenic (*Runx2*,* Alp,* and* Bglap*) (Figure 3G-J) and chondrogenic (*Coll0a-1*, *Col2a1,* and *Acan*) genes (Figure 3K-N). These observations suggested that PC1 was indispensable for osteochondral differentiation of PSPCs and cortical bone formation.

### 4. *Pkd1* deletion in *Ctsk^+^* PSPCs showed impaired fracture healing and diminished responsiveness to mechanical unloading

**Figure 4:**
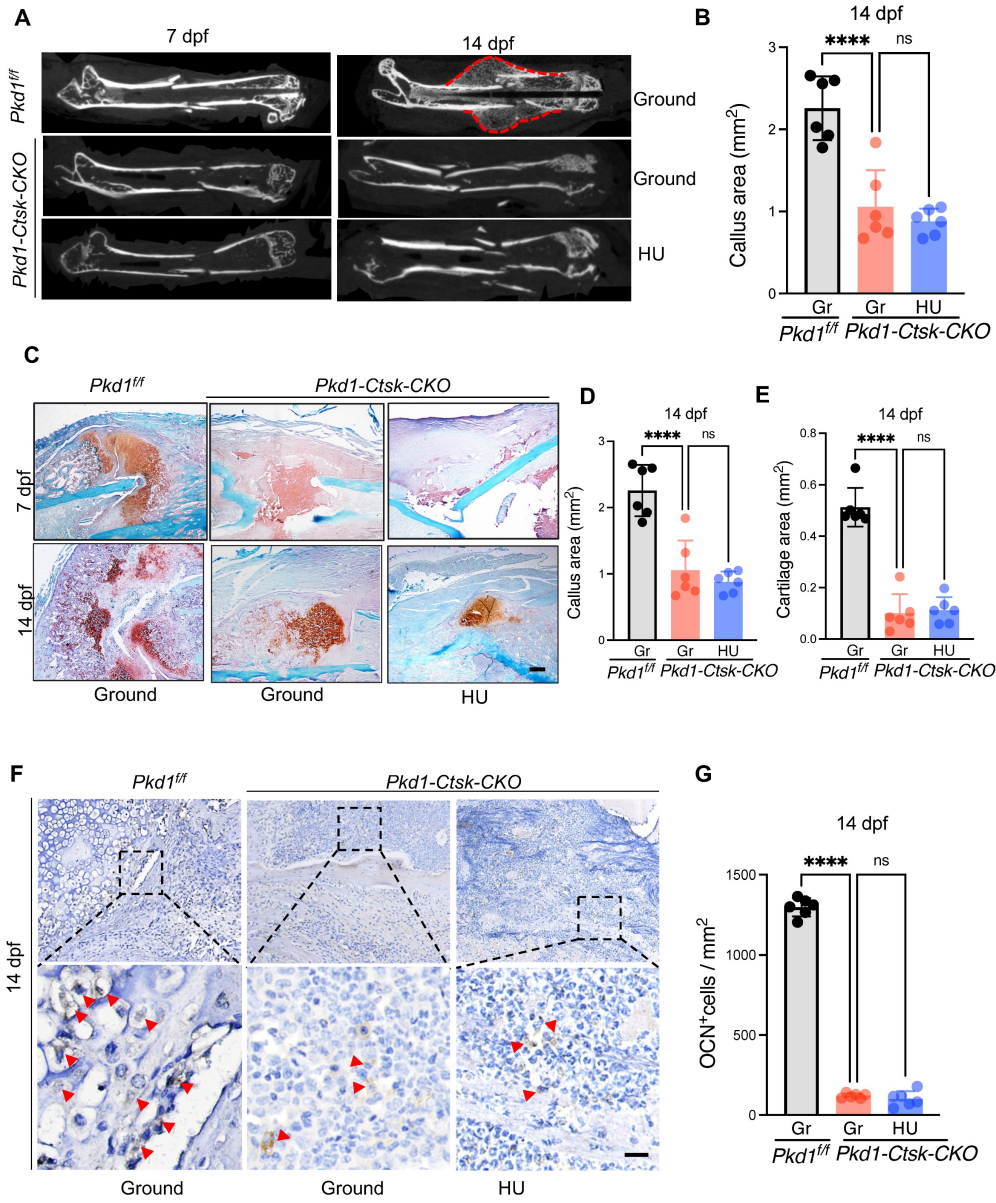
***Pkd1* deletion in *Ctsk^+^* PSPCs showed impaired fracture healing and diminished responsiveness to mechanical unloading. A** Representative micro-CT images of fractured femurs from *Pkd1^f/f^* and *Pkd1-Ctsk-CKO* mice treated with ground and HU at 7dpf and 14 dpf. **B** The callus index of fractured femurs from *Pkd1^f/f^* and *Pkd1-Ctsk-CKO* mice treated with ground and HU at 14 dpf. (n = 6).** C-E** Safranin O staining showed the cartilage callus formation and woven bone area from *Pkd1^f/f^* and *Pkd1-Ctsk-CKO* mice treated with ground and HU at 7 dpf and 14 dpf (n = 6). Scale bar indicates 200 μm. **F, G** Representative images of OCN immunohistochemical staining with analysis of number of osteoblasts (n = 6). Arrows indicate OCN positive cells. Data are shown as mean ± SD. *p < 0.05, ***p < 0.001 and **** p < 0.0001. ns, no significance.

*Pkd1-Ctsk-CKO* mice were subjected to transverse mid-diaphyseal femoral fracture to further test the role of *Pkd1* in *Ctsk^+^* PSPC function during fracture healing. Depletion of *Pkd1* in* Ctsk^+^* PSPCs diminished the callus size and ossification, as assessed by micro-CT (Figure 4A, B). We then performed the histological analysis in *Pkd1-Ctsk-CKO* mice to assess the chondrogenic and osteogenic potential of PSPCs in fracture repair. Compared to *WT* mice, reduced cartilaginous callus size, impaired bone formation, and fewer bone trabeculae were observed in *Pkd1-Ctsk-CKO* callus detected by Safranin O/Fast green staining and Masson staining (Figure 4C-E and Figure S5A-D). Similarly, lower numbers of OCN^+^ osteoblasts in the callus were found in the *Pkd1-Ctsk-CKO* group (Figure 4F, G). These data indicated that deletion of *Pkd1* in PSPCs had detrimental effects on bone healing.

Next, to assess whether PC1 mediates the effects of mechanical unloading on fracture healing and PSPC function, we performed a bone fracture in the HU model in *Pkd1-Ctsk-CKO* and *WT* mice. In contrast to the delayed fracture healing observed in *WT* mice under mechanical unloading, no significant distinction of callus index was observed in *Pkd1-Ctsk-CKO* mice that underwent ground and hindlimb unloading (Figure 4A-E). In agreement with this, no additional reduction was detected in the cartilaginous callus size and the number of OCN^+^ osteoblasts in *Pkd1-Ctsk-CKO* mice subjected to HU and fracture (Figure 4F, G). These findings suggested that the mechano-sensing capacity of the* Ctsk^+^* PSPCs was considerably diminished following *Pkd1* deletion, eventually resulting in resistance of *Pkd1-Ctsk-CKO* mice to unloading-associated fracture phenotype, providing strong evidence for the role of *Pkd1* in mechano-transduction in PSPCs during fracture healing.

### 5. PC1 C-terminal tail cleavage facilitates TAZ unclear translocation to enhance osteochondrogenesis of PSPCs

**Figure 5:**
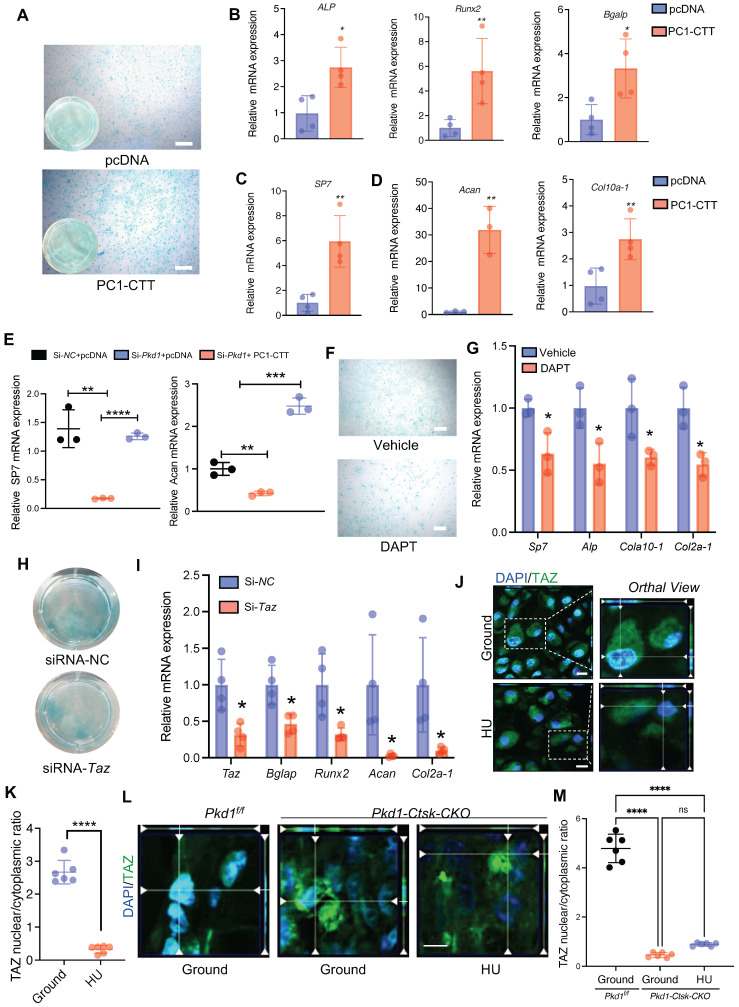
**PC1 regulates osteochondral differentiation potential of PSPCs via its C-terminal tail and downstream TAZ. A** Representative image of Alcian blue staining of PSPCs followed by chondrocyte differentiation induction with transfection of control or PC1-CTT plasmids. Scale bar indicates 100 μm. **B-D** qPCR analysis of osteogenic-related genes expression (B, C) and chondrogenic-related genes expression (D) in PSPCs transfected with control or PC1-CTT plasmids (n = 3-4). **E** qPCR analysis of *Sp7* and *Acan* in the indicated groups (n = 3). **F** Alcian blue staining of PSPCs followed by chondrocyte differentiation induction with 10uM DAPT or vehicle treatment. Scale bar indicates 100 μm. **G** qPCR analysis of *Sp7*, *Alp*, *Col10 a -1* and *Col2a-1* in the 10uM DAPT or vehicle treated groups (n = 3). **H** Alcian blue staining of PSPCs followed by chondrocyte differentiation induction with siRNA-*Taz* or siRNA-NC transfected. **I** qPCR analysis of *Taz*, *Bglap*, *Runx2*, *Acan-1* and *Col2a-1* of the PSPCs transfected with siRNA-*Taz* or siRNA-NC (n = 4). **J, K** Representative immunofluorescence staining images of TAZ (green) in PSPCs isolated from fracture femora in the ground and the HU treated group. Nuclei, DAPI (blue). Dotted squares indicate magnified areas. Scale bar indicates 10 μm.** L** Representative immunofluorescence staining images of TAZ (green) in PSPCs isolated from *Pkd1^ fl/fl^* mice and *Pkd1-Ctsk-CKO* mice femora. Nuclei, DAPI (blue). Scale bar indicates 5 μm. Data are presented as means ± SD. Unpaired t test. *p < 0.05, **p < 0.01, ***p < 0.001 and **** p < 0.0001.

The C-terminal tail (CTT) of PC1 transduces extracellular mechanical stimulation cues into the cell interior, facilitating transcriptional regulation 36, 42. It has been reported that replacing the full-length PC1 with PC1-CTT was sufficient for its binding to TAZ 36. Thus, we overexpressed PC1-CTT in PSPCs and tested the osteochondral differentiation ability. Compared to the vector control, the overexpression of PC1-CTT promoted osteochondral differentiation of PSPCs as evidenced by induced osteogenic (*ALP*, *Runx2*,* Sp7,* and* Bglap*) and chondrogenic (*Acan* and *Cola10-1*) gene expression*,* even in the absence of *Pkd1* (Figure 5A-E). To further test the impact of PC1-CTT on the osteochondral differentiation capability of PSPCs, we used DAPT, which functions as a γ-secretase inhibitor to restrict the cleavage and release of PC1-CTT 36. Compared to the controls, PSPCs treated with DAPT showed decreased osteochondrogenic-related gene expression and impaired differentiation of chondrocytes (Figure 5F, G). *In vitro* data showed that overexpression of PC1-CTT enhanced osteochondral differentiation of PSPCs in the absence of *Pkd1*. Also, DAPT, an inhibitor that restricts the cleavage and release of PC1-CTT, suppressed osteogenesis.

Previously, we reported that PC1 and TAZ form a functional complex to regulate osteogenesis 36, 43. Another study found that TAZ promoted osterix-positive osteoblast precursor expansion and differentiation during fracture healing 44. However, the effects of TAZ on the osteochondral differentiation potential of PSPCs have not been studied. We transfected *Taz* siRNA into PSPCs and analyzed their differentiation to osteoblasts and chondrocytes. The osteochondrogenic-related gene expression and the differentiation ability of PSPCs were suppressed by *Taz* knockdown (Figure 5H, I). Additionally, the mechanical unloading inhibited nuclear translocation of TAZ in the callus of *WT* mice with HU (Figure 5J, K). Interestingly, in the absence of *Pkd1* in PSPCs, the nuclear translocation of TAZ was also reduced in *Pkd1-Ctsk-CKO* mice (Figure 5L). These results suggested that PC1-CTT cleavage facilitated the nuclear translocation of TAZ, promoting the osteochondral differentiation potential of PSPCs.

### 6. A small molecule agent targeting PC1-TAZ axis alleviates unloading-related facture nonunion

**Figure 6:**
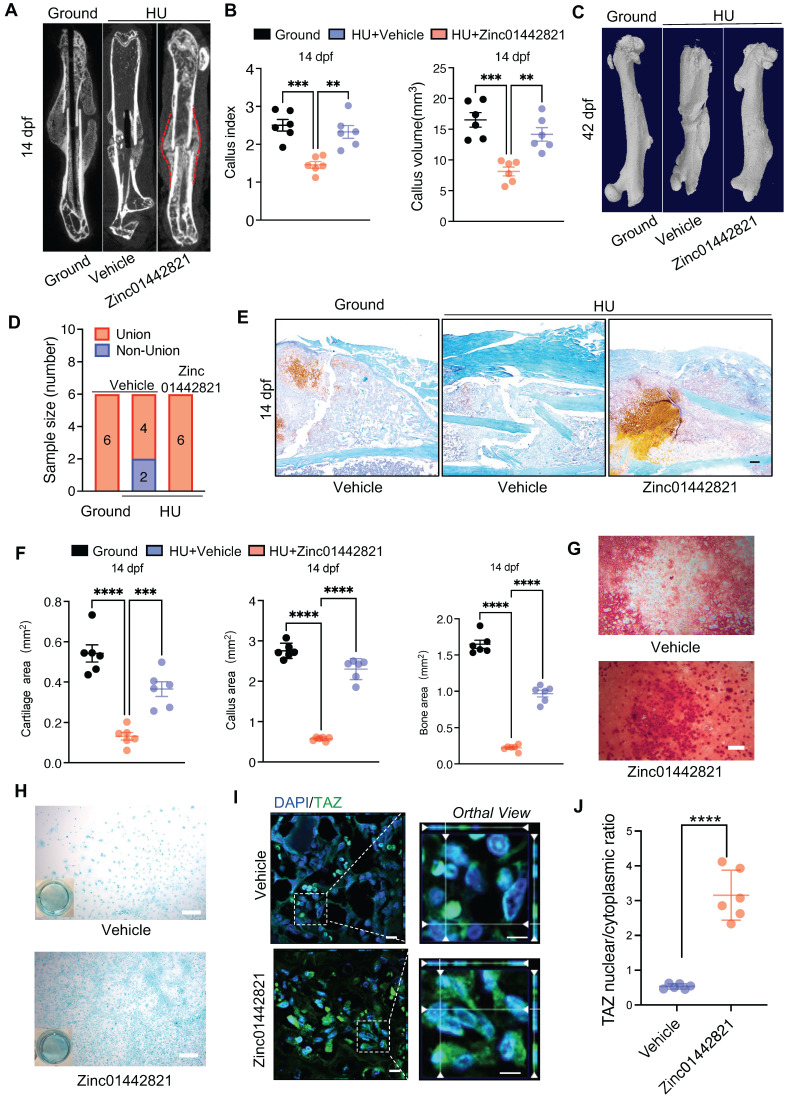
** Zinc01442821 promotes osteochondrogenesis of PSPCs and alleviates unloading-related fracture nonunion. A** Representative micro-CT images of fractured femurs from ground and HU mice treated with Vehicle or Zinc01442821 at 14 dpf. **B** The callus index of fractured femurs from indicated groups at 14 dpf (n = 6). **C, D** Representative 3D-μCT (C) and quantitative analysis (D) of images of fractured femurs from indicated groups at 42 dpf. **E, F** Safranin O staining showed the cartilage callus formation from indicated groups at 14 dpf. Scale bar indicates 100 μm. **G, H** Representative images of Alizarin Red staining (G) and Alcian blue staining (H) of PSPCs treated with vehicle or Zinc01442821. Scale bar indicates 100 μm. **I** Representative immunofluorescence staining images of TAZ (green) in PSPCs isolated from vehicle or Zinc01442821 mice femora. Nuclei, DAPI (blue). Dotted squares indicate magnified areas. Scale bar indicates 5 μm. **J** Quantification of the ratio of nuclear TAZ to cytoplasmic TAZ by Welch's t test (n = 6). Data are presented as means ± SD. Unpaired t test, **p < 0.01. **** p < 0.0001.

Our previous study identified a small molecule compound, Zinc01442821, which could increase bone mass by targeting the PC1-TAZ axis 36. Subsequently, we treated the bone fracture site in mice locally with Zinc01442821 for 42 days and investigated its therapeutic effect on fracture repair under unloading conditions. Micro-CT analysis demonstrated that Zinc01442821 increased bone density at the fracture site and improved mineralized callus formation relative to vehicle-treated controls under mechanical unloading conditions (Figure 6A). In the Zinc01442821-treated mice, hard callus was evident at the fracture site at 14 and 42 dpf (Figure 6A-D). Next, we performed SOFG staining to ascertain the size and structural characteristics of the fracture site. Compared to the vehicle-treated group, mice treated with Zinc01442821 exhibited a notable increase in the size and density of newly created woven bone (Figure 6E, F).

To investigate the impact of Zinc01442821 on osteochondrogenic capacity of PSPCs *in vitro*, we administered Zinc01442821 to PSPCs. Alizarin Red S staining revealed that the treatment increased osteogenic differentiation of PSPCs, as shown by nodule formation (Figure 6G). Furthermore, Alcian blue staining showed that Zinc01442821 promoted cartilaginous matrix formation of PSPCs (Figure 6H) and enhanced the nuclear translocation of TAZ (Figure 6I, J). These data demonstrated that administering Zinc01442821 can alleviate mechanical unloading-related bone delayed union or nonunion.

We also performed *in vitro* cell assays to validate the role of PC1 following Zinc01442821 treatment. We found that *Pkd1* knockdown blunted the positive effect of Zinc0144282 on PSPC osteogenic differentiation, as evidenced by the decreased nodule formation compared with siRNA-NC-treated controls (Figure S6A&B). This observation was consistent with the report that Zinc01442821 stimulated PC1- and TAZ-dependent osteogenesis, but *Pkd1*-deficient osteoblasts lost Zinc01442821 stimulation of intracellular calcium and TAZ activation 36. These data suggested that the therapeutic efficacy of Zinc01442821 was attenuated by *Pkd1* knockdown, highlighting the indispensable role of PC1 in Zinc01442821 treatment.

## Discussion

The absence of mechanical stimulation has a substantial detrimental impact on fracture healing 23, 45-48. Nevertheless, which cell type of PSPCs sense mechanical signals and the mechanism of the response to mechanical stimuli need to be clarified. Our study provided *in vitro* and *in vivo* evidence that the *Ctsk^+^* PSPCs can directly perceive mechanical stress and alter osteochondrogenesis through PC1. We found that mice with conditional depletion of *Pkd1* in *Ctsk^+^* PSPCs showed decreased sensitivity to mechanical stimuli, eventually resulting in compromised cortical homeostasis, impaired fracture healing, and a reduced sensitivity to mechanical unloading during fracture healing. Mechanistically, the C-terminal tail of PCI plays a crucial role in regulating the osteochondral differentiation of PSPCs by facilitating the translocation of TAZ to the nucleus. Moreover, the small molecule Zinc01442821, which targets the PC1-TAZ axis, improved the fracture healing during mechanical unloading.

The fracture healing process involves the participation of various cell types at the fracture site; hence, precise identification of the specific targeted cell is crucial. PSPCs are critical for skeletal healing, and PSPC dysfunction caused by stress conditions, like mechanical unloading, is emerging as a contributor to the pathogenesis of fracture nonunion. Nevertheless, due to the absence of specific markers to distinguish PSPCs *in vivo*, which PSPCs recognize fracture and how they initiate the repair process remain unknown. *Ctsk*, a specific biomarker of subsets of stem/progenitor cells, helps determine the crucial cells involved in skeletal diseases 22, 49. Here, we identified PSPCs carrying the* Ctsk* gene at the apex of the differentiation hierarchy. Single-cell profiling showed distinct and swift activation of *Ctsk^+^* PSPCs in the initial stage after bone fracture compared with other PSPCs. *Ctsk^+^* PSPCs demonstrated multipotency for differentiation into osteochondral cells, providing a cellular basis for osteoblastic and chondrogenic development, and were necessary for periosteal bone formation during bone regeneration and the establishment of normal cortical architecture.

Here, we showed that *Ctsk^+^* PSPCs were mechanical stimulus-sensitive, and unloading resulted in their dysfunction and impaired fracture healing. Besides, *Ctsk^+^* PSPCs sense mechanical signals, raising the possibility that PSPCs are attractive targets for drug and cellular therapy for nonunion fractures in mechanical unloading. We used scRNA-seq, fluorescent tracing in mice, and a series of experiments in genetic mouse models to elucidate the role of *Ctsk^+^* PSPCs in fracture repair under mechanical unloading. Unraveling their function and associated specific regulatory pathways is crucial for understanding nonunion fractures and developing therapeutic strategies.

*Prrx1* has been identified as a marker of periosteal stem cells. Single-cell profiling in this study showed *Prrx1* expression in *Ctsk^+^* PSPCs following fracture. A recent study found that periosteal *Prrx1*-lineage cells are important for fracture healing 8. Furthermore, another report proposed that the BMP-2/CXCL2 signaling regulates *Prrx1* expression during fracture 18. Although the importance of *Prrx1* cells in fracture repair is known, whether *Prrx1*-positive periosteal stem cells can sense mechanical stimuli, how they contribute to the process of bone fracture repair under mechanical unloading, and which molecular pathways mediate this function remain unclear. Future studies should investigate the bone phenotype of *Pkd1-Prrx1-CKO* mice and underlying molecular mechanisms of *Prrx1*-positive periosteal stem cells in response to mechanical stimuli.

PC1 encoded by *Pkd1* is widely expressed in various tissues and organs such as skin, kidneys, and musculoskeletal systems 31-36. Our previous studies have shown that PC1 is a key determinant of trabecular bone homeostasis 26, 31-35. However, the role of PC1 in fracture healing remains elusive. Our study showed that PC1 was abundantly expressed at the bone callus site after fracture and significantly decreased in *Ctsk^+^* PSPCs in response to mechanical unloading. However, it was dramatically increased under the FSS mechanical loading condition, suggesting that mechanical stimulus may regulate PSPC function by affecting the PC1 level. Herein, we provided evidence that PC1 regulated the osteochondral differentiation potential of *Ctsk^+^* PSPCs in response to various mechanical stimuli. Specific knockdown of *Pkd1* in* Ctsk^+^* PSPCs impaired PSPC function, resulting in abnormal cortical homeostasis and fracture repair. Moreover, *Pkd1-Ctsk-CKO* mice subjected to HU did not exhibit a further reduction in osteochondrogenic-related genes and bone healing ability. This suggested that PC1 serves as the primary mechanical sensor on *Ctsk^+^* PSPCs, and *Pkd1* depletion in *Ctsk^+^* PSPCs demonstrated a diminished ability to adapt to alterations in the mechanical milieu. Since PC1 is necessary for mechanical stimulation to regulate the differentiation ability of *Ctsk^+^* PSPCs and promotes bone healing, it could be used as a therapeutic target for fracture-related delayed union or nonunion.

Other mechanosensitive factors may also influence fracture healing under mechanical stimuli. However, in bone fracture healing, the mechanical stimuli may include shear stress, hydrostatic pressure, mechanical stretch, and tension. Our previous study reported that mechanical stretch stimulates nuclear translocation of the PC1-CTT/TAZ complex in multipotent mesenchymal cells, upregulating osteogenic gene expression mediated by Runx2 while simultaneously downregulating PPARγ- dependent adipogenic gene expression 36. Consistent with these observations, the *in vitro* data in the present study showed that overexpression of PC1-CTT enhanced osteochondral differentiation of PSPCs in the absence of Pkd1. In addition, DAPT restricted the cleavage and release of PC1-CTT, suppressing osteogenesis. Thus, our data indicated that PC1 and TAZ can form the PC1-CTT-TAZ complex; The cleavage of CTT from PC1 facilitates TAZ nuclear translocation and drives osteogenesis. We also found that mechanical unloading decreased nuclear translocation of TAZ, inhibiting osteochondrogenic-related genes and PSPC differentiation *in vitro*. Moreover, nuclear translocation of TAZ was also inhibited in *Pkd1-Ctsk-CKO* mice. The localized administration of the small molecule compound Zinc01442821, which specifically targets PC1-TAZ, exhibited augmentation in bone formation and restoration of bone regeneration in mechanical unloading conditions. These studies implied that TAZ activation may be required for osteochondral differentiation and bone healing. Further investigation is required to explore the precise molecular mechanism by which TAZ influences osteochondrogenesis and fracture repair.

Additionally, we found that the *Pkd1* expression was substantially downregulated with mechanical unloading. The geriatric population with a sedentary lifestyle may have reduced levels of mechanosensitive proteins (such as PC1). Hence, we hypothesize that the Zinc01442821 treatment might be helpful in the sedentary geriatric population to enhance fracture healing. Therapeutic options for bone fractures that cannot heal spontaneously are still limited. Current treatment options include transplantation of vascularized autologous tissues, distraction osteogenesis, and the induced membrane technique 50. The generation of periosteum-mimicking tissue has also become a novel strategy for critical-sized bone defects caused by trauma and bone tumor resection 51. Further studies are necessary to determine the effect and safety of Zinc01442821 treatment for bone defects.

One limitation of this study is the absence of models to investigate the effects of hydrostatic pressure, mechanical stretch, and tension on the PSPC function during fracture. In the future, we plan to construct *in vitro* and *in vivo* models to investigate the behavior of mechanosensitive cells (*Ctsk^+^* PSPCs) and their environment during the fracture healing.

In conclusion, our findings indicate that *Ctsk^+^* PSPCs can directly sense mechanical stress and manipulate osteochondrogenesis and bone healing via the PC1-TAZ axis, which may be a promising therapeutic target for promoting bone healing and other diseases associated with mechanical stress in mineralization tissues/organs.

## Methods

### Mice

The *Pkd1*-floxed mice were constructed using CRISPR/Cas9 technology in GemPharmatech Co., Ltd (China). The *YFP* reporter mice were purchased from the Jackson laboratory. The* CTSK-Cre* mice were provided by S. Kato (University of Tokyo). The *TRAP-Cre* mice was got from Cyagen Biosciences (China). The wild-type mice used in this study were purchased from Slack Jingda Company in Hunan Province. To obtained *Pkd1*-floxed mice were crossed with *CTSK-Cre* mice or *TRAP-Cre* mice separately to generate *Ctsk^+^* PSPCs-specific (*CTSK-Cre*) or osteoclast-specific (*TRAP-Cre*) *Pkd1* knockout mice, and the *Pkd1*-floxed littermates were used as controls. All mice were kept on a C57BL/6J background. In our experiments, standard housed mice were maintained in a temperature-controlled standard, specific pathogen-free facility (22 °C ± 1 °C) on a reverse 12 h light-dark cycle (07:00 to 19:00 light on). The mice were provided with standard food from Hunan SJA Laboratory Animal Company (China). Water was provided *ad libitum* and environmental enrichments. Control mice were selected as littermates that were matched for age and sex. The genotypes of the mice were identified through the utilization of PCR analyses on genomic DNA that was taken from mouse tail snips.

### Animal models

The mice were administered pentobarbital for anesthesia. The patella was laterally displaced in order to expose the femoral condyles. A 25-gauge regular bevel needle (BD, BioSciences) was then inserted to provide stability to the femur. A transverse fracture was intentionally induced at the mid-diaphysis using a bone saw. The muscles were brought back together, and the skin was stitched up using a 6/0 nylon suture. The mice were provided with appropriate thermal conditions throughout the duration of the process to mitigate the risk of hypothermia.

HU Animal Model: For HU model after unilateral femoral fracture, suspending the hind limbs mimicked the microgravity or disuse conditions as previously reported 5, 52, 53. One day after fracture modeling, 8 - to 10-week-old male (age-matched) genotype-matched mice were randomly separated into HU or ground controls. In the HU model, mice suspended in the cage and kept at 30° angle relative to the ground with the forelimbs accessible to the cage ground, while the hindlimbs were suspended in the air. The mice were inspected daily to prevent their hindlimbs from resting against the walls of the cage or connected to the cage ground. It was ensured that mice had freedom of movement and access to food and water.

For therapeutic effect evaluation of Zinc01442821 *in vivo*, unilateral femoral fracture mice in the treatment group were injected locally in fracture site with 100 mg/kg Zinc01442821 (Matrix Scientific) daily until time of sacrifice 36. The control mice administered a comparable volume of vehicle.

### Micro-CT analysis

Fractured bones were dissected at various time intervals and fixed for 24 hours with 4% paraformaldehyde, following scanned and analyzed with high-resolution micro-computed tomography (μCT) (Skyscan 1172, Bruker MicroCT, Kontich, Belgium) as previous reported 54, 55. The callus index (CI) of fractured bone was evaluated by calculating the ratio of the maximal diameter of the callus to the diameter of the adjacent diaphysis.

### Histochemistry and immunocytochemistry staining

Mouse femur samples was harvested after euthanasia, fixed in 4% paraformaldehyde at 4 °C for 12 hours, decalcified in 10% EDTA (pH 7.5) for 21 days and then embedded in paraffin. To obtain frozen sections, the fractured bones were fixed with 4% PFA, soaked in 30% sucrose and embedded in OCT compound. 5 μm sections were subjected to Safranin-O/Fast green (SOFG), Masson and hematoxylin and eosin (H&E) staining to quantify the cartilage area and woven bone area, as well as cortical bone thickness. For immunocytochemistry staining of OCN, antigen retrieval was performed, then the sections were incubated with primary antibodies (M137, 1:500, Takara) at 4 °C overnight. After rinsing three times, the sections were incubated with the appropriate secondary antibodies, following counterstaining the nucleus with hematoxylin (Sigma-Aldrich).

### Immunofluorescence staining

Immunofluorescence staining was conducted as previously described 54, 55. Briefly, femora were treated for antigen retrieval by digestion with 0.05% trypsin at 37 °C for 15 minutes, and stained with primary antibodies, including anti-Ctsk (ab19027, 1:200, Abcam), anti-GFP (ab6673, 1:200, Abcam), and anti-Ocn (M137, 1:500, Takara) overnight at 4 °C. Then, the slides were incubated at room temperature with secondary antibodies conjugated with fluorescence for an hour while keeping a dark environment. Nuclei were counterstained with 4',6-diamidino-2-phenylindole (DAPI; Sigma-Aldrich, St Louis, MO, USA). For immunofluorescent imaging, images were acquired with microscope (Leica).

### Primary PSPCs isolation and culture

PSPCs were isolated from tibias and intact femora from 4-week-old mice as described previously 8. Bone tissues were dissected, removed the adherent soft tissue and both ends of each bone carefully. Following the cut of both extremities of each bone, the bone marrow cavity was subjected to t rinse twice with 1 × PBS. Then, the rinsed bone (excluding bone marrow cells) was placed in a 10cm Petri dish and cultured in in α-MEM (Gibco) containing 10ng/ml basic fibroblast growth factor (bFGF) (Biotechne), 10% fetal bovine serum (FBS, Gibco) and 1% penicillin‐streptomycin (Gibco) (complete medium). All experiments were performed utilizing passage 2 (P2) periosteal stem/progenitor cells. All cells were cultured at a temperature of 37 ℃ within an environment that contained 5% carbon dioxide and maintained a level of humidity.

DAPT (Selleck) and Zinc01442821 (Matrix Scientific) were resuspended in DMSO and diluted in culture medium to the final concentration. To explore the effect of DAPT and Zinc on differentiation, DAPT (10 μM) and Zinc (10 μM) were added to cell culture medium after one day of induction and continued throughout the whole assay, with exchange of fresh medium every 3 days.

### Gene knockdown and overexpression

The siRNA-*NC*, siRNA-*Pkd1*, and siRNA-*Taz* were procured from RiboBio Co., Ltd. to suppress the expression of indigenous* Pkd1* and* Taz* genes. In order to overexpress* PC1-CTT*, plasmids were constructed to express MYC-tagged* PC1-CTT* (JTS scientific), or pcDNA (a blank vector). The transfection of siRNAs or plasmids was conducted using Lipofectamine RNAIMAX (Invitrogen) or Lipofectamine 2000 reagent (Invitrogen) in accordance with the instructions provided by the manufacturer. Subsequent interventions were conducted at a time interval of 6 hours following the transfections, after which the cells were gathered for further analysis.

### Horizontal shaker experiment

In order to induce hydrodynamics and administer shear stress, cells were performed by placing the 6/12 well plates onto a Boekel Rockerer shaker. The manipulation of the rocking angle and speed exerted influence over the amplitude of the shear stress. The rocking angle was adjusted to 6 degrees and the speed was set at 10 rocking motions per minute to generate a moderate level of shear stress. The shaker was positioned within a cell culture incubator that was kept at a temperature of 37 °C.

### Osteogenic differentiation assay

For osteoblastic differentiation, P2 periosteal stem/progenitor cells were seeded at a density of 3.0 × 10^4^/well and cultured with osteogenic-inducing medium (containing 10% FBS, 10 mM β-glycerophosphate, 0.05 mM ascorbic acid-2-phosphate and 0.1 μM dexamethasone) that was changed every 3 days. Then, cells were stained with 2% Alizarin Red S staining after 21 days of induction to detect cell matrix mineralization and calcium deposition.

### Chondrogenic differentiation assay

For chondrogenic differentiation, P2 PSPCs were cultured in chondrogenic medium (DMEM containing 10% FBS, 50 μg/mL ascorbic acid-2-phosphate, 0.1 μM dexamethasone, 50 mg/mL IvoS), which was changed every 3 days. The cell pellets were cultured for 21 days and then fixed in 4% paraformaldehyde fixative solution. To evaluate collagen deposition, the cells were stained with Alcian blue.

### qRT-PCR analysis

The isolation of total RNA from tissues and cell lysates was performed using Trizol reagent (Invitrogen). A reverse transcription kit (Accurate Biology) was utilized to generate single stranded cDNA from 1 μg of total RNA. For relative quantitative qRT-PCR, amplification reactions were set up in 10 μL reaction volumes containing the SYBR Green Premix *Pro Taq* HS qPCR Kit (ACCURATE BIOTECHNOLOGY (HUNAN) CO., LTD, ChangSha, China). Fold-alterations over controls were calculated using the relative quantification method of 2-ΔΔCt.

### Bioinformatics section

In the bioinformatics section, we established specific criteria for the inclusion and exclusion of datasets. The inclusion criteria required that the samples belonged to the species of mice, that the dataset was published within the past decade, and that the sample modelling method utilized femoral transection fracture with the receiving tissue being callus tissue. Conversely, the exclusion criteria were defined as any dataset that failed to meet the aforementioned inclusion criteria. Using these criteria, we conducted a screening of the Single-cell sequencing data obtained from the dataset GSE138689 in the GEO database. We performed dimensionality reduction and clustering using the Seurat version 3.1.1 56. For visualization of clusters, t-distributed Stochastic Neighbor Embedding (t-SNE) were generated using the same PCs 57. The log-normalized matrices were then loaded on SingleR R packages for cell type annotation, which based on correlating gene expression of reference cell types with single-cell expression. We obtained 4689 cells from control group and 2295 cells from fracture group for Single-cell analysis. To minimize the effects of batch effect and behavioral conditions on clustering, we used Harmony, an algorithm that projects cells into a shared embedding in which cells group by cell type rather than dataset-specific conditions, to aggregate all samples 58. Integrated expression matrix is then scaled and performed on principal component analysis for dimensional reduction. Then we chose top 50 principal components and cluster cells using the Seurat version 3.1.1 56. Seurat implements a graph-based clustering approach. Distances between the cells were calculated based on previously identified PCs. The DEGs in each cluster were used to perform GO enrichment analysis 59. The enriched GO terms were filtered by setting P-value cutoff to 0.05. Simplify function was performed to select the most significantly enriched terms.

### Statistical analysis

All data were statistically analyzed and visualized using GraphPad Prism 8.0 software (GraphPad Software Inc., La Jolla, CA). Data was presented as mean values ± standard deviation (SD) derived from a minimum of three independent experiments. A Shapiro-Wilk test for normality was performed on all datasets. The confirmation of homogeneity was achieved through the utilization of a test that compared variances. The t-test was used for two normally distributed samples with aligned variances, the approximate t-test or nonparametric rank sum test was used for uneven variances of two normally distributed samples. For multiple comparisons between more than two groups, we used the by one-way analysis of variance. The selection of the sample size for both *in vivo* and *in vitro* experiments was determined based on previous experience. The assignment of all samples was conducted in a random manner. The study did not use any initial exclusion criteria, and no animals or repetitions were excluded from the analysis. P value < 0.05 was considered statistically significant. Statistically significant differences are indicated as follows: * for p < 0.05 and ** for p < 0.01. *** for p < 0.001.

## Supplementary Material

Supplementary figures and table.

## Abbreviations

PC1: polycystin-1
PSPCs: periosteal stem/progenitor cells
Ctsk: cathepsin K
HU: hindlimb unloading
CI: callus index
scRNA-seq: single-cell RNA sequencing
GO: Gene Ontology
DEGs: differentially expressed genes
OCN: osteocalcin
ColII: type II collagen
FSS: fluid shear stress
SOFG: Safranin-O/Fast green
H&E: hematoxylin and eosin
μCT: micro-computed tomography
t-SNE: t-distributed Stochastic Neighbor Embedding
