# Supplementary material for: Mechanosensitive protein polycystin-1 promotes periosteal stem/progenitor cells osteochondral differentiation in fracture healing
Source: Theranostics. 2024 Apr 8;14(6):2544–59. doi: 10.7150/thno.93269 (PMC11024844; doi:10.7150/thno.93269)
Supplement: Supplementary file 1 — Supplementary figures and table. [file thnov14p2544s1.pdf]

Table S1. The number and percentage of PSPCs-1 & 2 in control and fracture group.

Cluster	Control	Fracture
PSPC-1	192 (4.09%)	227 (9.89%)
PSPC-2	79 (1.68%)	105 (4.58%)

Figure S1

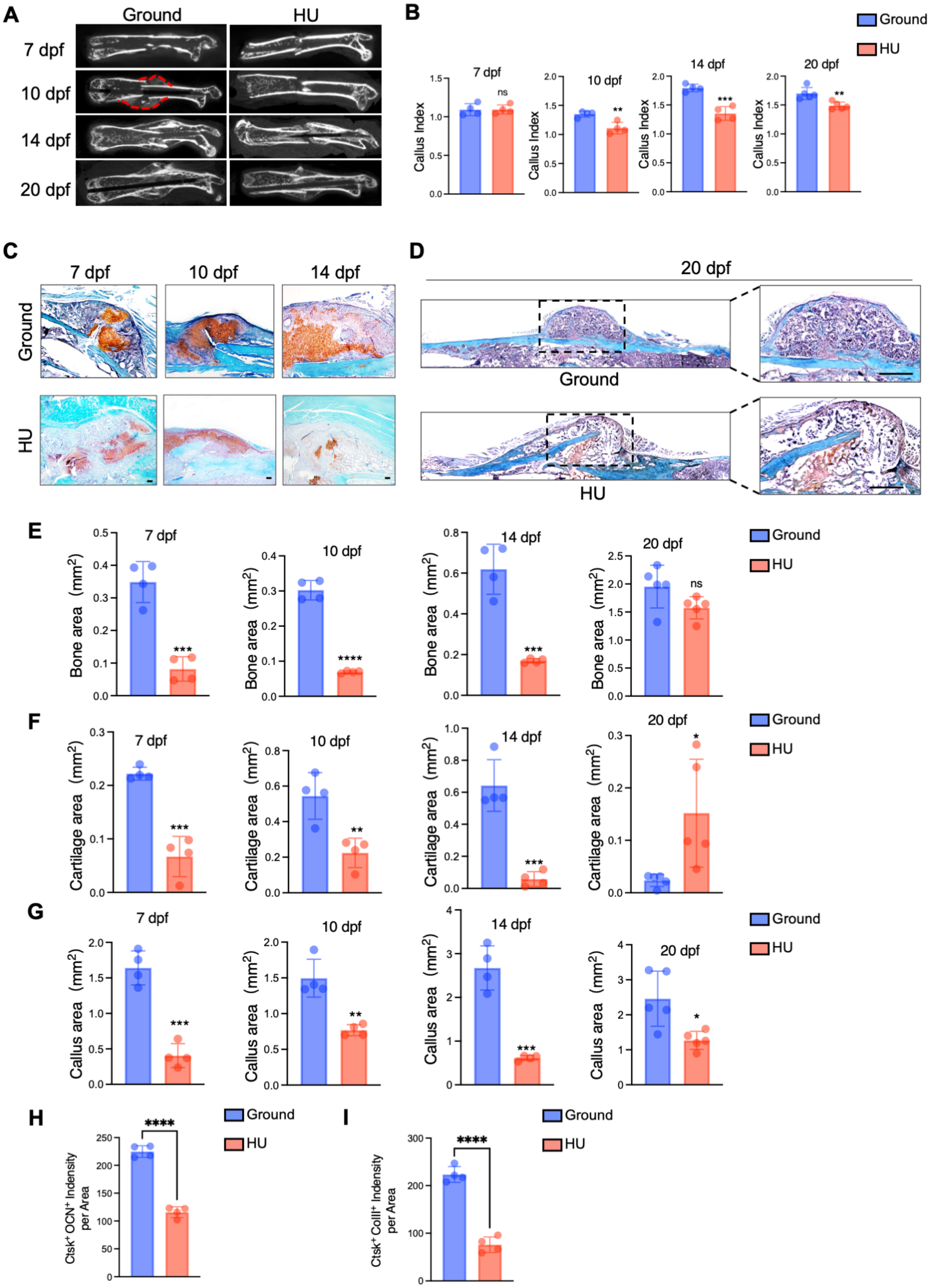
A
Ground
HU
7 dpf
10 dpf
14 dpf
20 dpf
B
Ground
HU
Callus Index
ns
**
***
C
D
Ground
HU
E
Bone area (mm2)
****
F
Cartilage area (mm2)
*
G
Callus area (mm2)
H
Ctsk+ OCN+ Indensity per Area
I
Ctsk+ CollI+ Indensity per Area

Figure S1 (Related to Figure 1). Mechanical unloading results in delayed fusion. **A** Representative micro-CT images of fractured femurs from ground and HU treated mice at 7, 10, 14 dpf and 20 dpf (n = 4). **B** The callus index of fractured femurs from ground and HU treated mice at 7, 10, 14 dpf and 20 dpf (n = 4-5). **C** Safranin O staining showed the cartilage callus formation from ground and HU treated mice fractured femurs at 7, 10 and 14 dpf (n = 4-5). Scale bar indicates 100 μm. **D** Safranin O staining showed the cartilage callus formation from ground and HU treated mice fractured femurs at 20 dpf (n = 4-5). Dotted squares indicate magnified areas. Scale bar indicates 200 μm. **E, F** The bone area (E) and cartilage area (F) of fractured femurs from ground and HU treated mice at 7, 10, 14 dpf and 20 dpf (n = 4-5). **G** The callus area of fractured femurs from ground and HU treated mice at 7, 10, 14 dpf and 20 dpf (n = 4-5). **H** Quantification of Ctsk and OCN immunofluorescence of fracture callus at 14 days post-fracture in *Ctsk-Cre; YFP*$^{+/+}$ mice (n = 4). **I** Quantification of Ctsk and CoLll immunofluorescence of fracture callus at 14 days post-fracture in *Ctsk-Cre; YFP*$^{+/+}$ mice (n = 4). Data are presented as means ± SD. Unpaired t test. *p < 0.05, ***p < 0.001 and **** p < 0.0001.

Figure S2

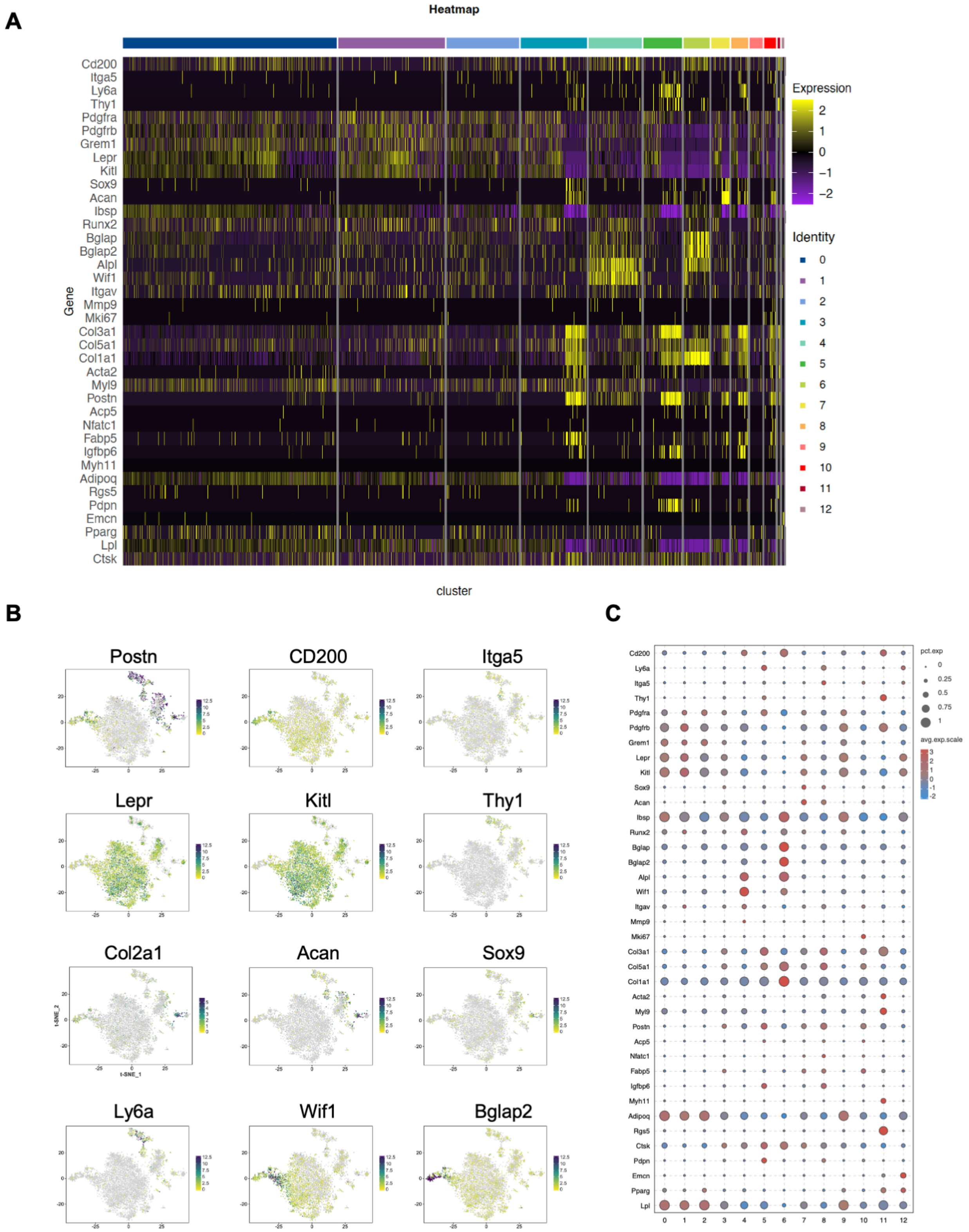

Figure S2 (Related to Figure 1). *Ctsk*$^{+}$ PSPCs in fractured callus. A Heatmap showing the relative expression levels (row-wide Z score) of the significant marker

genes for each cluster (rows) across cells in the 13 clusters (columns) identified and color-coded from mice fracture models (control group and fracture group). **B** Expression of marker genes for cell populations highlighted on t-SNE. **C** Bubble chart showing the expression of feature genes for each cluster.

Figure S3

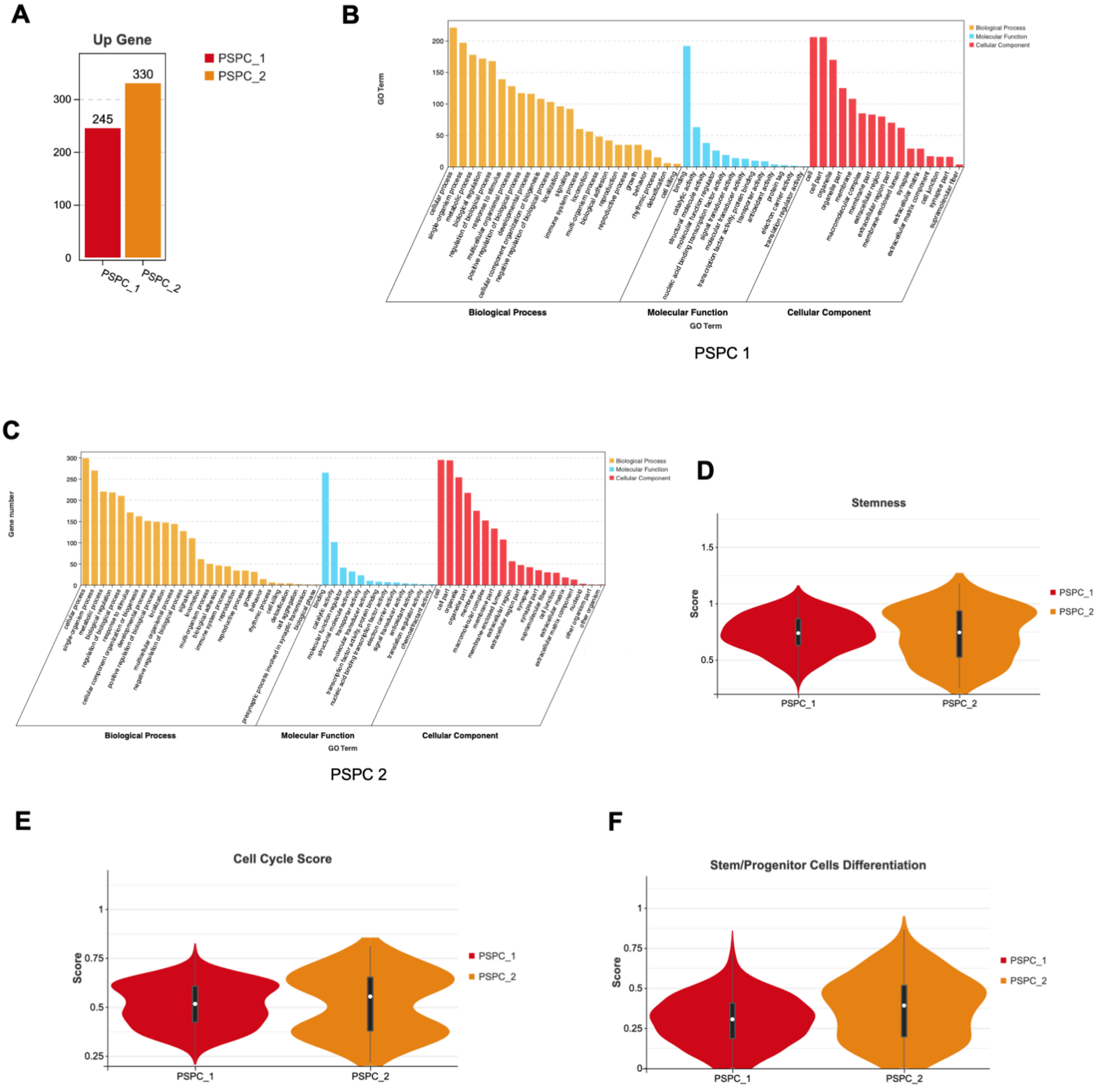

Figure S3 (Related to Figure 1). The comparison of PSPCs-1 & 2 in fractured callus. A Stacked bar chart showing the up gene of PSPCs-1 & 2 within callus tissue quantified at 7 days post-fracture. **B, C** GO analysis of differentially expressed genes in PSPCs-1 & 2. **D-F** Violin plots demonstrating the score of stemness (**D**), cell cycle (**E**) and stem /progenitor cells differentiation (**F**) between PSPC-1 and 2.

Figure S4

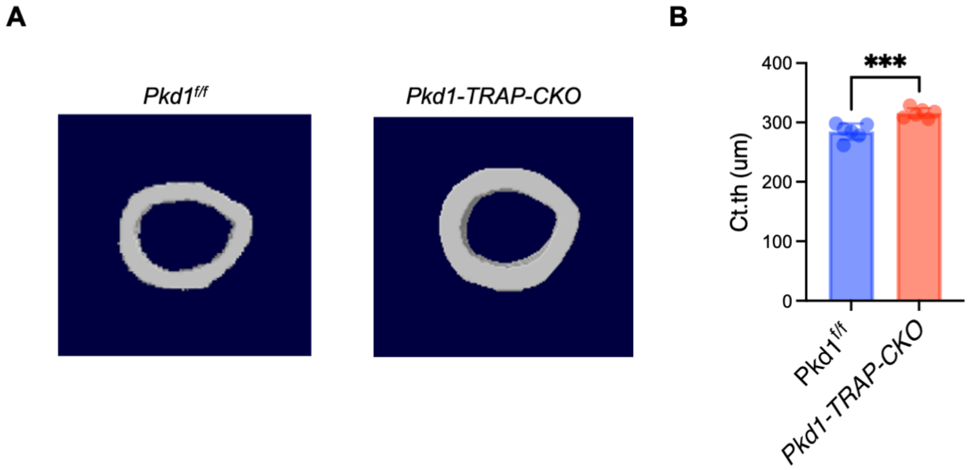

Figure S4 (Related to Figure 3). *Pkd1* deletion in *Trap*$^+$ osteoclasts. A, B μCT images in femurs from 8-week-old male *Pkd1-Trap-CKO* mice (A) and quantitative analysis of the indicated parameters in *Pkd1-Trap-CKO* mice (B), respectively (n = 6).

Figure S5

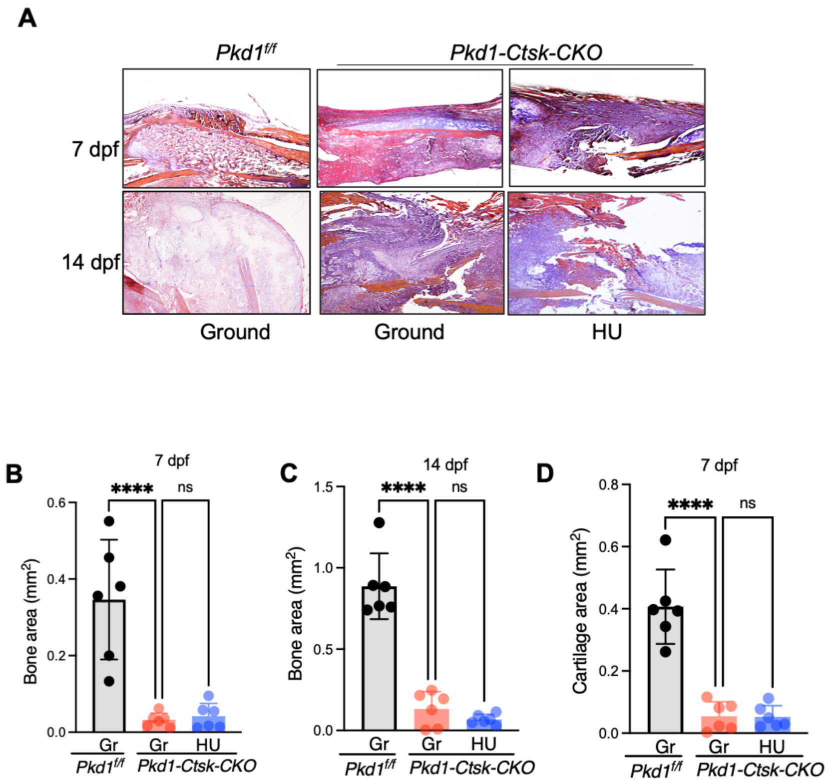

Figure S5 (Related to Figure 4). *Pkd1* deletion leads to impaired fracture healing. **A** Masson staining showed the woven bone area and cartilage area from *Pkd1*$^{f/f}$ and *Pkd1-Ctsk-CKO* mice treated with ground and HU at 7 dpf and 14 dpf (n = 6). **B, C** Quantification of the bone area (B, C) from *Pkd1*$^{f/f}$ and *Pkd1-Ctsk-CKO* mice treated with ground and HU at 7 dpf and 14 dpf (n = 6). **D** Quantification of the cartilage area from *Pkd1*$^{f/f}$ and *Pkd1-Ctsk-CKO* mice treated with ground and HU at 7 dpf (n = 6). Scale bar indicates 100 µm. Data are presented as means ± SD. **** $p < 0.0001$. ns, no significance.

Figure S6

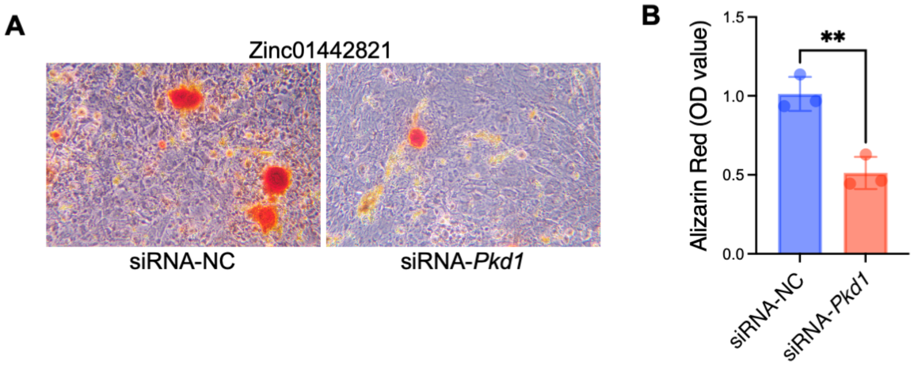

Figure S6 (Related to Figure 6). ***Pkd1*** **deletion leads to attenuated therapeutic efficacy of Zinc01442821. A-B** Representative images of Alizarin Red S staining (A) and quantification of staining (B) of PSPCs transfected with *Pkd1* siRNA or siRNA-NC and with Zinc01442821 treatment.
